# ^18^F-PI-2620 Tau PET is associated with cognitive and motor impairment in Lewy body disease

**DOI:** 10.1093/braincomms/fcae458

**Published:** 2024-12-19

**Authors:** Joseph R Winer, Hillary Vossler, Christina B Young, Viktorija Smith, America Romero, Marian Shahid-Besanti, Carla Abdelnour, Edward N Wilson, David Anders, Aimara Pacheco Morales, Katrin I Andreasson, Maya V Yutsis, Victor W Henderson, Guido A Davidzon, Elizabeth C Mormino, Kathleen L Poston

**Affiliations:** Department of Neurology and Neurological Sciences, Stanford University School of Medicine, Stanford, CA 94304, USA; Department of Neurology and Neurological Sciences, Stanford University School of Medicine, Stanford, CA 94304, USA; Department of Neurology and Neurological Sciences, Stanford University School of Medicine, Stanford, CA 94304, USA; Department of Neurology and Neurological Sciences, Stanford University School of Medicine, Stanford, CA 94304, USA; Department of Neurology and Neurological Sciences, Stanford University School of Medicine, Stanford, CA 94304, USA; Department of Neurology and Neurological Sciences, Stanford University School of Medicine, Stanford, CA 94304, USA; Department of Neurology and Neurological Sciences, Stanford University School of Medicine, Stanford, CA 94304, USA; Department of Neurology and Neurological Sciences, Stanford University School of Medicine, Stanford, CA 94304, USA; Department of Radiology, Stanford University School of Medicine, Stanford, CA 94304, USA; Department of Radiology, Stanford University School of Medicine, Stanford, CA 94304, USA; Department of Neurology and Neurological Sciences, Stanford University School of Medicine, Stanford, CA 94304, USA; Department of Neurology and Neurological Sciences, Stanford University School of Medicine, Stanford, CA 94304, USA; Department of Neurology and Neurological Sciences, Stanford University School of Medicine, Stanford, CA 94304, USA; Department of Epidemiology and Population Health, Stanford University, Stanford, CA 94304, USA; Department of Radiology, Stanford University School of Medicine, Stanford, CA 94304, USA; Department of Neurology and Neurological Sciences, Stanford University School of Medicine, Stanford, CA 94304, USA; Wu Tsai Neuroscience Institute, Stanford University, Stanford, CA 94304, USA; Department of Neurology and Neurological Sciences, Stanford University School of Medicine, Stanford, CA 94304, USA; Wu Tsai Neuroscience Institute, Stanford University, Stanford, CA 94304, USA

**Keywords:** Lewy body disease, tau PET imaging, mixed pathology

## Abstract

Co-pathology is frequent in Lewy body disease, which includes clinical diagnoses of both Parkinson’s disease and dementia with Lewy bodies. Measuring concomitant pathology *in vivo* can improve clinical and research diagnoses and prediction of cognitive trajectories. Tau PET imaging may serve a dual role in Lewy body disease by measuring cortical tau aggregation as well as assessing dopaminergic loss attributed to binding to neuromelanin within substantia nigra. We sought to characterize ^18^F-PI-2620, a next generation PET tracer, in individuals with Lewy body disease. We recruited 141 participants for ^18^F-PI-2620 PET scans from the Stanford Alzheimer’s Disease Research Center and the Stanford Aging and Memory Study, most of whom also had β-amyloid status available (139/141) from PET or cerebrospinal fluid. We compared ^18^F-PI-2620 uptake within entorhinal cortex, inferior temporal cortex, precuneus and lingual gyrus, as well as substantia nigra, across participants with Lewy body disease [Parkinson’s disease (*n* = 29), dementia with Lewy bodies (*n* = 14)] and Alzheimer’s disease (*n* = 28), in addition to cognitively unimpaired healthy older adults (*n* = 70). Mean bilateral signal was extracted from cortical regions of interest in ^18^F-PI-2620 standard uptake value ratio (inferior cerebellar grey reference) images normalized to template space. A subset of participants received cognitive testing and/or the Movement Disorders Society Unified Parkinson’s Disease Rating Scale Part III motor exam (off medication). ^18^F-PI-2620 uptake was low overall in Lewy body disease and correlated with β-amyloid PET in temporal lobe regions and precuneus. Moreover, inferior temporal ^18^F-PI-2620 uptake was significantly elevated in β-amyloid positive relative to β-amyloid negative participants with Lewy body disease. Temporal lobe ^18^F-PI-2620 signal was not associated with memory in Lewy body disease, but uptake within precuneus and lingual gyrus was associated with worse executive function and attention/working memory performance. Finally, substantia nigra ^18^F-PI-2620 signal was significantly reduced in participants with Parkinson’s disease, and lower substantia nigra signal was associated with greater motor impairment. These findings suggest that although levels are lower than in Alzheimer’s disease, small elevations in cortical tau are associated with cognitive function in Lewy body disease relevant domains, and that reduced ^18^F-PI-2620 binding in substantia nigra may represent loss of dopaminergic neurons. Cortical tau and neuromelanin binding within substantia nigra represent two unique signals in the same PET image that may be informative in the context of cognitive and motor deficits, respectively, in Lewy body disease.

## Teaser

Winer *et al*. report on binding patterns of the ^18^F-PI-2620 tau PET tracer in participants with Lewy body disease relative to unimpaired controls and participants with Alzheimer’s disease. Cortical uptake is related to amyloid deposition and worse cognitive performance, while signal in substantia nigra is associated with motor impairment.

## Graphical Abstract

**Graphical abstract:**
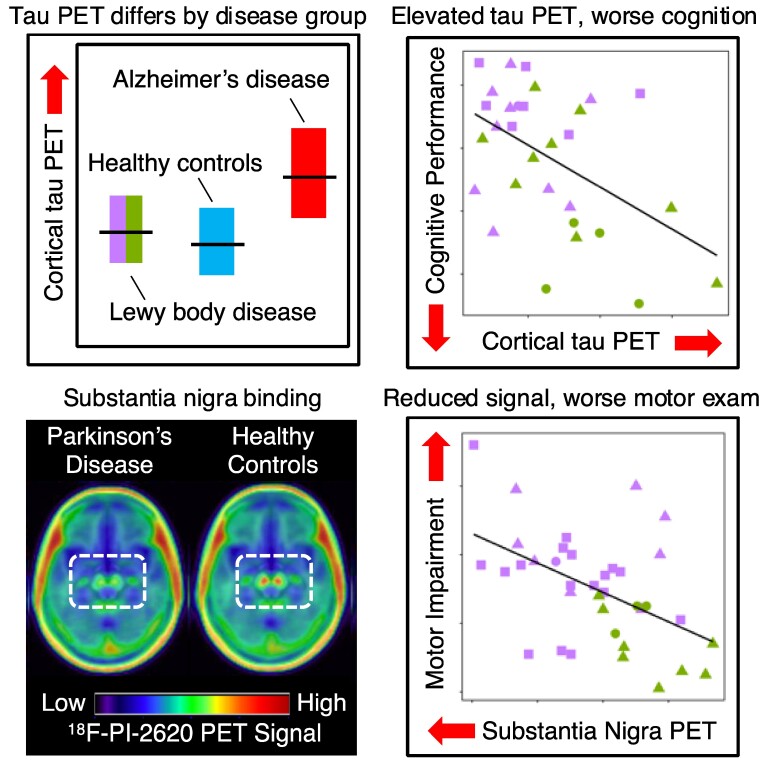

## Introduction

Lewy body disease (LBD) is a progressive neurodegenerative disorder characterized neuropathologically by intracellular aggregates of alpha-synuclein, typically occurring together with neuronal loss affecting the dopaminergic nigrostriatal pathway. LBD is clinically diagnosed as Parkinson’s disease when initially manifesting as bradykinesia and rigidity, or as dementia with Lewy bodies (DLB) when cognitive impairment occurs before the onset of motor symptoms.^1,2^ While limbic and neocortical alpha-synuclein are thought to drive cognitive impairment in LBD,^3-5^ concomitant Alzheimer’s disease pathology [β-amyloid (Aβ) plaques and tau neurofibrillary tangles] is frequently seen at autopsy in individuals with LBD. Crucially, Alzheimer’s disease co-pathology may contribute to cognitive decline, and the presence of Aβ and tau in postmortem studies of LBD has been linked to greater memory deficits,^6^ earlier age of dementia,^7,8^ and shorter duration to death.^9^ However, the prevalence of Alzheimer’s disease co-pathology postmortem in patients diagnosed with LBD varies significantly among studies, with reports of concomitant Alzheimer’s disease (at levels expected to cause cognitive impairment) in Parkinson’s disease dementia patients varying between 25% and 60%,^5,9^ as well as findings of at least low levels of Alzheimer’s disease pathology (Thal Aβ phase 1 or greater) in 60–90% of LBD cases.^10,11^ This high prevalence of mixed LBD and Alzheimer’s disease pathology highlights the need for *in vivo* measures of neuropathology, which can be leveraged to characterize individuals’ pathological and cognitive trajectories.

In the last decade, beginning with the development of ^18^F-flortaucipir and more recently with the advent of radioligands such as ^18^F-PI-2620, tau PET imaging has enabled imaging of tau neurofibrillary tangles in people with Alzheimer’s disease as well as co-morbid tau in people with LBD and other neurodegenerative diseases. Initial studies utilizing ^18^F-flortaucipir found that cortical tau burden in Parkinson’s disease is at levels similar to healthy controls and does not differentiate participants with Parkinson’s disease who are cognitively normal and participants with Parkinson’s disease and cognitive impairment.^12,13^ Studies that have included individuals with DLB have reported that ^18^F-flortaucipir uptake is elevated in DLB relative to healthy controls, but that cortical uptake is low in comparison to participants with Alzheimer’s disease.^14-18^ Findings from studies using other tau PET tracers (^18^F-florzolotau, ^18^F-MK6240) in LBD are similar.^19,20^ Beyond assessing the magnitude of tangle burden, another important question that can be addressed with tau PET is whether topographical patterns of cortical tau differ in individuals on the LBD clinical spectrum compared to the typical Braak staging pattern in Alzheimer’s disease. Some studies, though not all, have found that on a group level participants with DLB show elevated ^18^F-flortaucipir binding in posterior temporoparietal and occipital cortex relative to healthy controls,^15,18^ as well as one report of increased signal in sensorimotor cortex.^16^ These inconsistent findings across the LBD tau PET literature are likely due to (i) small sample sizes (*n* < 30 participants^12-15,17,19,20^) with varied groupings of impairment across the LBD clinical spectrum, (ii) a lack of matching Aβ burden to controls, which is likely to influence tau aggregation in LBD and (iii) a small number of individuals with elevated tau PET driving group comparison effects, despite a majority of LBD scans displaying low cortical signal.^14,17^

The binding pattern of tau PET radioligands in the brain reflects more than just tau tangles.^21,22^ While the ‘off-target’ binding profile for these tracers remains imperfectly understood, it is clear that they bind to regions dense in neuromelanin, particularly in substantia nigra, where neuromelanin is thought to reflect dopaminergic neuronal counts.^23-25^ Studies utilizing this property of ^18^F-flortaucipir have shown that binding in substantia nigra is reduced in individuals with Parkinson’s disease relative to healthy controls,^12,25^ and lower ^18^F-flortaucipir signal in substantia nigra is associated with greater motor impairment.^18^^18^F-PI-2620 similarly binds to neuromelanin in substantia nigra,^22,26^ but it is not yet known whether ^18^F-PI-2620 signal may carry information about dopaminergic neuron loss or motor impairment.

We investigated patterns of ^18^F-PI-2620 uptake in participants with LBD, including those with Parkinson’s disease and DLB, compared to participants with Alzheimer’s disease and cognitively unimpaired healthy older individuals. We sought to determine whether patterns of ^18^F-PI-2620 tau PET binding in LBD are associated with Aβ, and if they differ in individuals with LBD compared to Alzheimer’s disease and healthy older adults. We further hypothesized that in LBD greater ^18^F-PI-2620 PET signal in cortex would be related to worse cognitive impairment, and less ^18^F-PI-2620 PET signal in substantia nigra would be related to greater motor impairment.

## Materials and methods

### Participants

We studied 141 participants from the Stanford Alzheimer’s Disease Research Center and Stanford Aging and Memory Study who underwent a research ^18^F-PI-2620 tau PET scan. All participants underwent diagnostic adjudication at a multidisciplinary consensus meeting, which included a panel of neurologists, neuropsychologists and research staff. Parkinson’s disease was diagnosed using the UK Brain Bank criteria and required bradykinesia with muscle rigidity and/or rest tremor.^2^ Participants were further categorized as having Parkinson’s disease with mild cognitive impairment (PD-MCI) if they had a cognitive complaint and objective impairment on comprehensive cognitive testing using MDS Level II criteria^27^ without substantial impact on functional activities. Parkinson’s disease dementia was defined as cognitive impairment severe enough to interfere with activities of daily living^28^ as determined by clinical history and the Clinical Dementia Rating.^29^ Mild cognitive impairment due to Lewy bodies (MCI-LB) and DLB were defined according to published criteria.^1,30^ All participants diagnosed clinically with mild cognitive impairment due to Alzheimer’s disease or Alzheimer’s disease dementia met criteria for National Institutes of Health Alzheimer’s Disease Diagnostic Guidelines^31,32^ and were confirmed to have biomarker evidence of Alzheimer’s disease using Aβ PET or CSF Aβ assay. All healthy controls (HC) were older adults with no parkinsonian symptoms, normal neurological examination, and performance on comprehensive neuropsychological testing that was normal for age and sex. Study participants were grouped into four clinical aetiological categories: Parkinson’s disease (Parkinson’s disease without cognitive impairment, Parkinson’s disease with mild cognitive impairment, and Parkinson’s disease dementia), DLB (MCI-LB and DLB), Alzheimer’s disease (mild cognitive impairment due to Alzheimer’s disease and Alzheimer’s disease dementia) and HC. The Stanford Institutional Review Board approved this study, and all study participants provided written informed consent.

### Tau PET imaging

PET images were acquired using a simultaneous time-of-flight (TOF) enabled PET/MRI scanner (Signa 3T, GE Healthcare). Following a 5 to 10 mCi injection of ^18^F-PI-2620, data were collected either 45 to 75 min or 60 to 90 min post-injection. PET images were reconstructed using TOF optimized subset expectation maximization with 3 iterations, 28 subsets and 2.78 × 1.17 × 1.17 mm voxel size. Corrections were applied for detector deadtime, scatter, randoms, detector normalization and radioisotope decay. Attenuation correction was performed with ZTE MRI for MR attenuation correction.^33^ PET data were reconstructed into 5-min frames and these frames were realigned and summed. ^18^F-PI-2620 standard uptake volume ratio (SUVR) was calculated for each PET image using an inferior cerebellum reference region defined on participants’ structural MRI (high-resolution T1-weighted spoiled gradient recalled echo scan [repetition time = 7.664 ms, echo time = 3.09 ms, inversion time = 400 ms, flip angle = 11, 1.2 × 1.1 × 1.1 mm]) by the spatially unbiased atlas template toolbox in MATLAB.^34^

Because the ^18^F-PI-2620 dataset consisted of a mix across two different acquisition protocols (45 to 75 and 60 to 90 min), we adjusted 60 to 90 min data to be on a 45 to 75 min SUVR scale using methods described by Pontocorvo *et al*.^35^ In this method, a slope is calculated for every voxel using all possible frame data, and this slope is used to interpolate the estimated value at 60 min (the midpoint of the 45 to 75 min window) using the value at 75 min (the midpoint of the 60 to 90 window). This method was validated on a subset of 15 participants with coverage across both 45 to 75 and 60 to 90 time windows. This acquisition timing adjustment method is described in full in Young *et al*.^36^

Following these adjustments, SUVR images were warped to MNI152 space in SPM12 (Wellcome Trust Centre for Neuroimaging) using each participant’s T1-weighted MRI. Mean SUVR within FreeSurfer v7 regions of interest (ROIs) from the Desikan aparc + aseg atlas were extracted from each participant’s Montreal Neurological Institute-space image using the normalized probability Desikan-Killiany atlas.^37^ Analyses focused on four cortical regions: two bilateral ROIs in the temporal lobe typically utilized in capturing typical Alzheimer’s disease progression (entorhinal cortex and inferior temporal cortex) and two bilateral ROIs previously shown to be elevated in LBD (precuneus, lingual gyrus).^14,15^ For each cortical region, tau positivity was defined as 2 SD greater than the mean of the Aβ-HC group.^38,39^ To examine ^18^F-PI-2620 signal in substantia nigra, we extracted mean SUVR from each participant’s MNI-space SUVR image within a bilateral substantia nigra ROI from an MNI-space version of the Talairach atlas.^40^ The substantia nigra mask was generated using WFU PickAtlas in SPM12.

### β-Amyloid data

^18^F-florbetaben Aβ PET scanning was completed in 89 participants using a simultaneous time-of-flight-enabled PET/MRI scanner (Signa 3T, GE Healthcare). Data were acquired between 90 and 110 min following an 8.1mCi injection of ^18^F-Florbetaben. PET data were reconstructed into 5-min frames using standard methods with ZTE or Dixon MR imaging for MR attenuation correction. Five-minute frame data were realigned and summed. FreeSurfer v7 ROIs from the Desikan aparc + aseg atlas were defined on each participant’s structural MRI. Native space FreeSurfer ROIs were used to extract intensity values from the co-registered summed PET data. SUVR was calculated for a global cortical ROI using a whole cerebellum reference region, centiloids were calculated using the equation from Royse *et al*.^41^ (centiloid = 157.15 × SUVR − 151.87). Centiloid values were only used in analyses if the ^18^F-florbetaben PET scan was collected within 3 years of the ^18^F-PI-2620 PET scan (included centiloid values for 83 participants were collected 0.70 ± 0.92 years from ^18^F-PI-2620 scan). Aβ status (±) was determined for the 89 participants with ^18^F-florbetaben PET imaging using a cutoff of 18 centiloids.^41^

CSF samples were collected in 41 participants by lumbar puncture at 8 or 9 a.m. CSF was stored in 1.0 or 0.5 mL aliquots at −80°C. A single aliquot for each participant was used to measure Aβ1–42 and Aβ1–40 using the fully automated Lumipulse *G* system (Fujirebio US, Inc., Malvern, PA, USA) in a single-batch analysis.^42,43^ CSF were collected 1.23 ± 1.3 years from ^18^F-PI-2620 scan. Using a larger dataset of 153 participants, a 2-cluster Gaussian mixture modelling approach was used to define a Aβ42/Aβ40 cutoff of 0.0752 (based off the 0.5 probability of belonging to the Aβ+ distribution).^42^

Nine additional participants that did not undergo amyloid PET or lumbar puncture as part of this study had Aβ status derived from pre-existing clinical CSF or PET which provided only dichotomous Aβ status. Aβ data were included in analyses if an individual was (i) Aβ+ at any time before or <3 years after the ^18^F-PI-2620 PET scan or (ii) Aβ− within 3 years of the ^18^F-PI-2620 PET scan. Aβ data were not available for one participant with Parkinson’s disease and one participant with MCI-LB.

### Cognitive and motor data

A subset of *n* = 30 individuals with LBD completed a neuropsychological battery (described in detail in Shahid *et al*.^44^) within two years of their ^18^F-PI-2620 PET scan. Our analyses focused on three cognitive domains: (i) episodic memory, (ii) executive function and (iii) attention/working memory. For each neuropsychological test, z-scores were calculated using means and standard deviations of baseline visit data from 236 cognitively unimpaired participants in the Stanford Alzheimer’s Disease Research Center. Domain-specific composite scores were created by averaging z-scores within each cognitive domain. The memory composite consisted of Craft story delayed recall, Benson Figure delayed recall, Hopkins Verbal Learning Test—Revised delayed recall and Free and Cued Selective Reminding Test free delayed recall (requiring at least 2 out of 4 scores). Executive function was comprised of phonemic fluency, semantic fluency, clock drawing, Trails B duration, Stroop colour-word and digit symbol (requiring at least 4 out of 6 scores). Attention/working memory was comprised of Trails A duration, digits forward, digits backward, Stroop dot counting, Stroop word reading and Letter Number Sequencing (requiring at least 4 out of 6 scores). We also investigated associations with the Montreal Cognitive Assessment (MoCA) total score as a measure of global cognition.

Motor impairment severity was evaluated using the Movement Disorder Society-sponsored revision of the Unified Parkinson’s Disease Rating Scale Part III (MDS-UPDRS-III) in the off-medication state (‘OFF’), defined according to published protocols (≥ 48 h off extended release dopamine agonists, selective monoamine oxidase inhibitors, and long-acting levodopa, and ≥12 h off short-acting dopamine agonists and levodopa^45^). MDS-UPDRS-III OFF scores were available from *n* = 35 participants with LBD. Since the time between ^18^F-PI-2620 PET scan and MDS-UPDRS exam varied across participants (median −1.8; range −4.6 to 2.3 years from PET scan to MDS-UPDRS), we used this interval as a covariate in regression models.

### Statistical analysis

Mean ^18^F-PI-2620 differences between groups in cortical ROIs and substantia nigra were assessed using nonparametric Kruskal–Wallis tests, followed by *post hoc* Dunn’s tests.

Associations between ^18^F-PI-2620 binding in cortical ROIs and ^18^F-florbetaben centiloids were tested with linear regressions which adjusted for age at ^18^F-PI-2620 scan, sex and time interval between ^18^F-florbetaben scan and ^18^F-PI-2620 scan, with ^18^F-florbetaben data restricted to scans collected within 3 years of the ^18^F-PI-2620 scan. Associations between ^18^F-PI-2620 binding in cortical ROIs and cognitive domain scores were tested with linear regressions, which adjusted for age at PET scan and sex, with cognitive data restricted to assessments taking place within 2 years of the PET scan. Associations between substantia nigra ^18^F-PI-2620 binding and MDS-UPDRS-III scores were tested with linear regressions which adjusted for age at PET scan, sex and the time interval between MDS-UPDRS exam and PET scan. No multiple comparisons correction was performed. All statistical analyses were performed in R version 4.2.1 (R Foundation for Statistical Computing). We considered two-sided *P* < 0.05 to be statistically significant. We used the STROBE cross-sectional checklist when writing our report.^46^

## Results

Participant demographics are summarized in Table 1. The Parkinson’s disease group (*n* = 29) was comprised of 18 individuals without cognitive impairment, 10 individuals with Parkinson’s disease with mild cognitive impairment and one individual with Parkinson’s disease dementia; the DLB group (*n* = 14) consisted of 10 individuals with MCI-LB and four individuals with DLB; and the Alzheimer’s disease group (*n* = 28) was made up of 12 individuals with MCI and 16 individuals with dementia. There were no significant differences in age across Parkinson’s disease, DLB, Alzheimer’s disease or HC groups (*P* = 0.59). Sex distribution differed across groups (*P* = 0.02) such that there were fewer female individuals with Parkinson’s disease and DLB relative to individuals with Alzheimer’s disease and the HC group.

**Table 1:** Participant demographics

	All	Parkinson’s disease	DLB	HC	Alzheimer’s disease
*N*	141	29	14	70	28
Age	72.3 ± 8.2	73.3 ± 8.2	73.9 ± 9.2	71.5 ± 7.7	72.6 ± 8.9
*N*, % female	72 (51)	10 (34)	4 (29)	44 (63)	14 (50)
*N*, % MCI	32 (23)	10 (34)	10 (71)		12 (42)
*N*, % dementia	21 (15)	1 (3)	4 (29)		16 (57)
*N*, % Aβ+	75 (53)	11 (38)	7 (50)	29 (41)	28 (100)
Years of education	16.9 ± 2.2	17.3 ± 1.7	16.9 ± 2.7	16.8 ± 2.2	17.1 ± 2.2
Years since diagnosis		10.5 ± 4.4	5.0 ± 2.4		5.6 ± 2.2
MoCA	25.2 ± 4.1	26.5 ± 3.2	22.8 ± 5.6	26.8 ± 2.3	21.1 ± 3.8
MMSE				29.3 ± 0.8	
MDS-UPDRS-III (OFF)		36.5 ± 14.6	15.2 ± 9.3		
APOE genotype					
ɛ2/ɛ3	8	0	1	6	1
ɛ2/ɛ4	2	0	0	2	0
ɛ3/ɛ3	64	23	7	39	6
ɛ3/ɛ4	24	4	5	15	7
ɛ4/ɛ4	8	1	1	5	3
Missing APOE data	15	1	0	3	11

Two participants (one cognitively normal participant with Parkinson’s disease, one participant with mild cognitive impairment due to Lewy body disease) did not have β-amyloid status. Six percent of participants were missing years of education.

Fourteen percent of participants were missing years since diagnosis (not including healthy controls), 35% of individuals were missing MoCA (primarily healthy controls), and 11% of individuals were missing APOE genotype.

Fifty-four percent of healthy controls were assessed with MMSE instead of MoCA, MMSE is reported for these individuals (*N* = 32).

DLB, dementia with Lewy bodies; HC, healthy controls; MCI, mild cognitive impairment; Aβ+, β-amyloid positive; MoCA, Montreal Cognitive Assessment; MMSE, Mini Mental State Exam, MDS-UPDRS-III (OFF), Movement Disorders Society Universal Parkinson’s Disease Rating Scale Part III (Off medication).

### Associations between β-amyloid PET and ^18^F-PI-2620 PET

**Figure 1:**
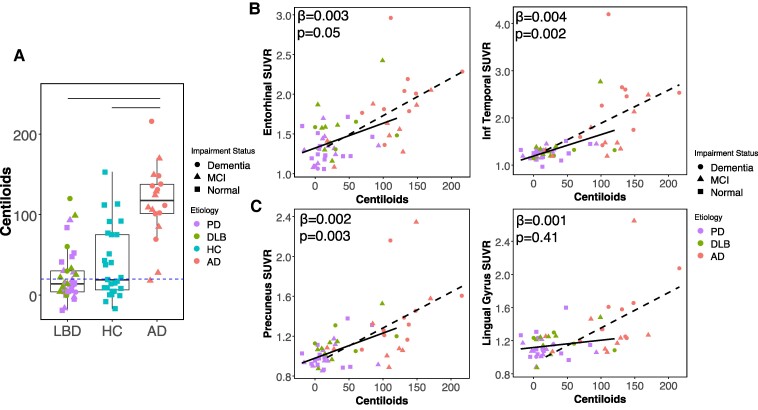
**Associations between ^18^F-PI-2620 tau PET in cortical regions and ^18^F-florbetaben β-amyloid PET.** (**A**) Boxplots show median and interquartile range for each group, dashed blue line indicates β-amyloid positivity threshold of 18 centiloids. Kruskal–Wallis test indicates significant difference in centiloids across groups. Top horizontal lines indicate significant *post hoc* Dunn’s tests (*P* < 0.05). ^18^F-florbetaben centiloids are lower in Lewy body disease relative to other Alzheimer’s disease but do not differ from healthy control participants. (**B**) In temporal lobe regions, ^18^F-PI-2620 tau PET SUVR is associated with ^18^F-florbetaben centiloids in Lewy body disease. (**C**) In participants with Lewy body disease, precuneus ^18^F-PI-2620 SUVR is associated with ^18^F-florbetaben centiloids, but lingual gyrus ^18^F-PI-2620 SUVR is not. Estimates and *P*-values reflect linear regressions which included age and sex as co-variates. Slopes are shown for both Lewy body disease (solid line) and Alzheimer’s disease (dashed line for visual comparison), statistics are shown only for Lewy body disease group. SUVR, standardized uptake value ratio; PD, Parkinson’s disease; DLB, dementia with Lewy bodies; AD, Alzheimer’s disease; HC, healthy controls.

Eighteen (42%) LBD participants [11 (38%) Parkinson’s disease, 7 (50%) DLB] were Aβ+, while 29 (41%) of HC participants were Aβ+. In the subset of individuals with ^18^F-florbetaben PET, centiloids did not differ between Parkinson’s disease, DLB and HC (Fig. 1A, *P* > 0.12), but were elevated in Alzheimer’s disease relative to all other aetiology groups (*P* < 0.001). Centiloids were significantly lower in Aβ+ LBD relative to Alzheimer’s disease (*P* = 0.04) but did not differ from Aβ+ HC (*P* = 0.53).

Across the combined LBD group (Parkinson’s disease + DLB), we examined the association between ^18^F-florbetaben PET and ^18^F-PI-2620 PET in cortical ROIs to determine whether ^18^F-PI-2620 PET binding is associated with Aβ deposition. In participants with LBD, centiloids were associated with entorhinal (Fig. 1B, unstandardized estimate ± S.E., 0.01 ± 0.002, *P* = 0.05) and inferior temporal ^18^F-PI-2620 SUVR (0.004 ± 0.001, *P* = 0.002). Centiloids were additionally associated with precuneus ^18^F-PI-2620 SUVR (Fig. 1C, 0.002 ± 0.001, *P* = 0.003), but not lingual gyrus ^18^F-PI-2620 SUVR (0.001 ± 0.001, *P* = 0.41).

### Associations between ^18^F-PI-2620 PET, β-amyloid status and clinical aetiology

**Figure 2:**
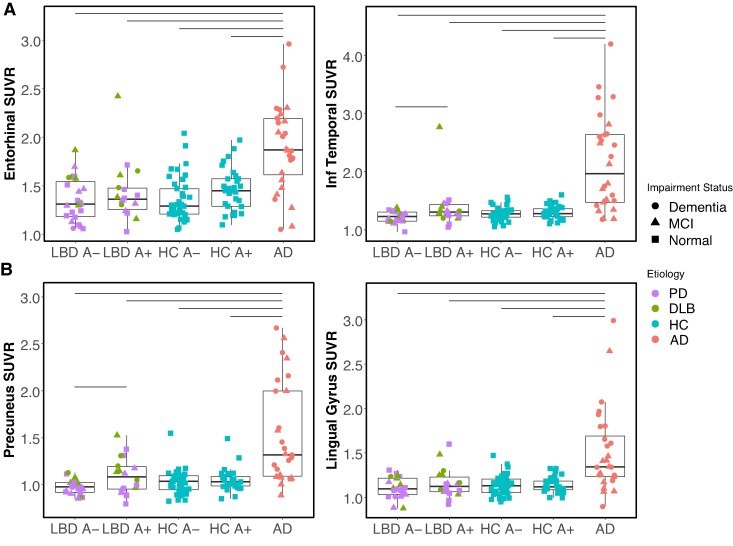
**Cortical ^18^F-PI-2620 tau PET in Lewy body disease, Alzheimer’s disease, and healthy control participants stratified by β-amyloid status.** Boxplots show median and interquartile range for each group. In all comparisons, Kruskal–Wallis test indicates significant difference in SUVRs across groups. Horizontal lines indicate significant *post hoc* Dunn’s tests (*P* < 0.05). (**A**) Left, ^18^F-PI-2620 SUVR in entorhinal cortex does not differ between β-amyloid positive and β-amyloid negative Lewy body disease groups but is marginally elevated in β-amyloid positive relative to β-amyloid negative healthy controls. Right, in inferior temporal cortex, ^18^F-PI-2620 SUVR is elevated in the β-amyloid positive relative to the β-amyloid negative Lewy body disease group. ^18^F-PI-2620 SUVR is significantly higher in Alzheimer’s disease relative to all other groups in both temporal lobe regions. (**B**) Left, in precuneus, ^18^F-PI-2620 SUVR is elevated in β-amyloid positive Lewy body disease group compared to the β-amyloid negative Lewy body disease group. The β-amyloid positive healthy control group also had marginally higher SUVR than β-amyloid negative Lewy body disease group. ^18^F-PI-2620 SUVR is elevated in the Alzheimer’s disease group relative to all other groups in both precuneus and lingual gyrus. One outlier is omitted from the precuneus plot, a participant with Alzheimer’s disease with 4.22 SUVR. SUVR, standardized uptake value ratio; PD, Parkinson’s disease; DLB, dementia with Lewy bodies; AD, Alzheimer’s disease; HC, healthy controls, A+/−, β-amyloid positive/negative.

Since we observed Aβ associations with cortical ^18^F-PI-2620 SUVR in LBD, we grouped individuals with Parkinson’s disease and DLB into one LBD group and also stratified LBD and HC participants by Aβ status (+/−). (Figure 2A). Entorhinal ^18^F-PI-2620 uptake was greater in Aβ+ HC relative to Aβ− HC (*P* = 0.05) but did not differ in between Aβ+ and Aβ− LBD groups (*P* = 0.48). There were no other significant differences observed between groups (*P*’s > 0.13). Entorhinal ^18^F-PI-2620 signal was significantly higher in the Alzheimer’s disease group relative to all other groups. In inferior temporal lobe, Alzheimer’s disease was again elevated relative to all other groups (*P*’s ≤ 0.001). In addition, Aβ+ LBD was elevated relative to Aβ− LBD (*P*’s < 0.04), but not Aβ− HC (*P* = 0.31). When removing one Aβ+ LBD individual with the highest inferior temporal SUVR, the difference from the Aβ− LBD group was slightly attenuated (*P* = 0.08).

In posterior ROIs, individuals with Alzheimer’s disease again demonstrated ^18^F-PI-2620 binding that was elevated relative to all other groups, in both precuneus and lingual gyrus (Fig. 2B, *P*’s ≤ 0.002). In precuneus, mean ^18^F-PI-2620 SUVR was significantly elevated in Aβ+ LBD compared to Aβ− LBD (*P* = 0.03). Aβ+ HC also had marginally higher signal than Aβ− LBD (*P* = 0.08), but there were no other significant differences between groups (*P*’s > 0.31). In lingual gyrus, there were no other significant differences between groups (*P*’s > 0.36).

Based on a tau positivity cutoff of 2 SD greater than the mean of the Aβ+ HC group, 2 (5%) LBD participants, 5 (7%) HC participants and 17 (60%) Alzheimer’s disease participants were tau positive in entorhinal cortex; 3 (7%) LBD, 4 (6%) HC and 20 (71%) Alzheimer’s disease in inferior temporal cortex; 5 (12%) LBD, 3 (4%) HC and 16 (57%) Alzheimer’s disease in precuneus; and 3 (7%) LBD, 2 (3%) HC and 13 (46%) Alzheimer’s disease in lingual gyrus.

### Associations between ^18^F-PI-2620 PET and cognition

**Figure 3:**
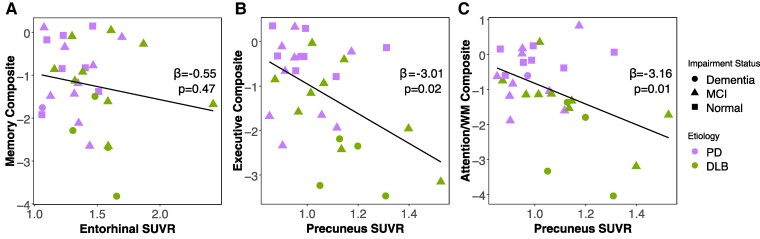
**Cortical ^18^F-PI-2620 tau PET associations with cognitive performance in Lewy body disease.** Scatterplots show associations between ^18^F-PI-2620 SUVR in cortical regions and cognitive domain composite scores for individuals with Lewy body disease. (**A**) Entorhinal cortex ^18^F-PI-2620 SUVR is not associated with memory performance. (**B**) Greater precuneus ^18^F-PI-2620 SUVR is associated with worse executive function performance. (**C**) Greater precuneus ^18^F-PI-2620 SUVR is associated with worse attention and working memory performance. Estimates and *P*-values reflect linear regressions which included age and sex as co-variates. SUVR, standardized uptake value ratio; PD, Parkinson’s disease; DLB, dementia with Lewy bodies.

First, we sought to determine whether greater ^18^F-PI-2620 binding in temporal cortical tau regions was associated with cognitive impairment in individuals with LBD. Episodic memory scores were not associated with ^18^F-PI-2620 binding in entorhinal cortex (Fig. 3A, −0.55 ± 0.75, *P* = 0.47) or inferior temporal cortex (0.04 ± 0.66, *P* = 0.95). There was no significant evidence of an association between ^18^F-PI-2620 SUVRs in entorhinal cortex and inferior temporal lobe with executive function or attention/working memory composite scores, or MoCA total score (*P*’s > 0.11).

Second, we investigated whether ^18^F-PI-2620 binding in posterior cortical regions, specifically precuneus and lingual gyrus, was associated with cognition. Here, we found greater ^18^F-PI-2620 uptake in precuneus was associated with worse executive function scores (−3.01 ± 1.16, *P* = 0.02) and with worse attention/working memory composite scores (−3.16 ± 1.18, *P* = 0.01). Further, greater ^18^F-PI-2620 SUVR in lingual gyrus showed a marginal association with worse executive function (Fig. 3B, −2.71 ± 1.52, *P* = 0.09) and a significant association with worse attention/working memory (Fig. 3C, −3.50 ± 1.51, *P* = 0.03). Memory composite score and MoCA total score were not significantly associated with ^18^F-PI-2620 SUVR in either posterior ROI (*P*’s > 0.32).

### Associations between substantia nigra ^18^F-PI-2620 PET, clinical aetiology, and motor impairment

**Figure 4:**
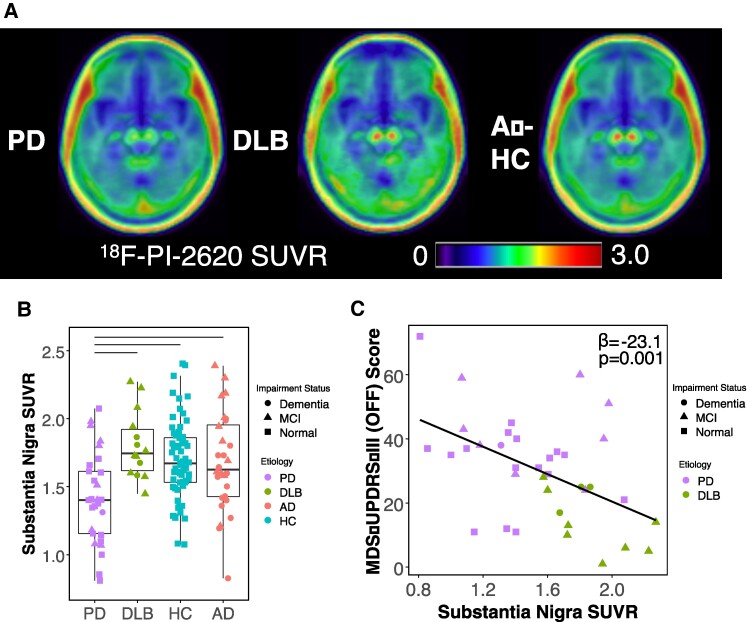
**Substantia nigra ^18^F-PI-2620 PET uptake differs by clinical aetiology and is associated with severity of motor impairment in Lewy body disease**. (**A**) Group mean images show ^18^F-PI-2620 PET SUVR in substantia nigra across participants with Parkinson’s disease, participants with dementia with Lewy bodies and β-amyloid negative healthy control participants. (**B**) Boxplot shows median and interquartile range mean ^18^F-PI-2620 PET SUVR within substantia nigra across aetiological groups, horizontal lines indicate significant Dunn’s tests (*P* < 0.05). Signal is reduced in Parkinson’s disease group relative to all other groups. One outlier is omitted, a healthy control participant with 3.78 SUVR. (**C**) In participants with Lewy body disease, Movement Disorders Society Universal Parkinson’s Disease Rating Scale III motor exam (administered in an off medication state) is negatively associated with mean substantia nigra SUVR, such that individuals with reduced SUVR demonstrate greater motor impairment. Estimate and *P*-value reflect a linear regression which included age, sex, and time interval. SUVR, standardized uptake value ratio; PD, Parkinson’s disease; DLB, dementia with Lewy bodies; AD, Alzheimer’s disease; HC, healthy controls; Aβ-, β-amyloid negative; MDS-UPDRS-III (OFF), Movement Disorders Society Universal Parkinson’s Disease Rating Scale Part III (Off Medication).

Given the predominance of motor symptoms in Parkinson’s disease, we separated out Parkinson’s disease and DLB when examining group differences in substantia nigra. ^18^F-PI-2620 binding in substantia nigra was reduced in the Parkinson’s disease group relative to all other groups (Fig. 4A and B, *P*’s < 0.01) but did not differ among other groups (*P*’s > 0.20). In participants with LBD, substantia nigra ^18^F-PI-2620 signal was negatively associated with MDS-UPDRS-III OFF scores, such that reduced substantia nigra SUVR was related to greater severity of motor impairment (Fig. 4C, −23.1 ± 6.58, *P* = 0.001).

## Discussion

This cross-sectional study of *n* = 41 individuals across the LBD spectrum (Parkinson’s disease + DLB) represents to our knowledge the first examination of ^18^F-PI-2620 tau PET in LBD and is one of the largest studies of tau PET imaging in LBD using any tau PET radiotracer. We investigated patterns of ^18^F-PI-2620 tau PET binding relative to individuals with Alzheimer’s disease and healthy older adults, associations between cortical ^18^F-PI-2620 PET uptake and cognition, and the relationship between ^18^F-PI-2620 PET signal in substantia nigra and motor impairment. We found that in participants with LBD, ^18^F-PI-2620 SUVR is correlated with ^18^F-florbetaben Aβ PET and is low throughout temporal and posterior cortical regions. ^18^F-PI-2620 SUVRs in precuneus and lingual gyrus, regions previously reported to reflect a pattern of elevated tau PET uptake in LBD, were associated with worse performance in tests of executive function and attention/working memory. Finally, substantia nigra SUVR was reduced in participants with Parkinson’s disease, and lower substantia nigra SUVR was associated with more severe motor impairment.

It is increasingly appreciated that most older individuals have multiple brain pathologies regardless of cognitive status or diagnosis. Recent postmortem studies suggest that the presence of α-synuclein aggregates with at least a low level of concomitant Alzheimer’s disease pathology (Aβ and/or tau) is especially common, representing 60–90% of LBD cases.^10,11^ Seemingly in contrast to the postmortem literature, studies of tau PET in LBD (the majority of which use ^18^F-flortaucipir) have demonstrated that tau PET SUVRs in participants with LBD are generally within the range of healthy control participants,^12,13,18^ with a small percentage of cognitively impaired LBD participants showing elevated binding within temporal and posterior cortical regions, though not at levels as high as those typically seen in Alzheimer’s disease.^14,15,17-19^ We found that Aβ+ individuals with LBD had higher inferior temporal lobe tau PET compared to Aβ− individuals with LBD. The inferior temporal lobe is an important region in the progression of Alzheimer’s disease, considered to mark the spread of tau pathology outside of medial temporal lobe.^47,48^ Thus these findings suggest that tau in Aβ+ LBD participants may follow a spatial pattern similar to early stages of Alzheimer’s disease, despite this group having levels of ^18^F-PI-2620 considerably lower than the Alzheimer’s disease participants included in the present study. Interestingly, one detailed postmortem study found that tau burden across the brain was greater in individuals with Alzheimer’s disease without LBD relative to concomitant LBD and Alzheimer’s disease.^49^ One feasible explanation for the gap in LBD tau burden between the postmortem findings and tau PET findings is that in many individuals with LBD, concomitant tau is present but not at a level detectable with tau PET, which may not be sensitive to tau deposition in the early Alzheimer’s disease Braak stages.^50^ There may be several reasons why Aβ+ participants with LBD have lower tau burden relative to participants with Alzheimer’s disease, despite similar time since clinical diagnosis. First, Aβ accumulation may start later in the disease process in LBD. Second, Aβ may occur at a slower rate. Third, there was a small proportion of LBD participants with dementia (5 out of 43) in the present study. The inclusion of a large fraction of cognitively normal participants with Parkinson’s disease could be contributing to the negative results. Longitudinal data will be necessary to explore these possibilities.

Some prior studies have suggested that patterns of tau PET uptake in LBD may be distinct from the typical Alzheimer’s disease Braak staging pattern, with higher signal in posterior temporoparietal and occipital cortex and reduced medial temporal signal relative to unimpaired controls,^15-17^ although other studies have not observed this pattern.^12,13,18^ Our data did not support the hypothesis of a unique spatial pattern of tau deposition in LBD relative to Alzheimer’s disease, with no significant difference in precuneus and lingual gyrus SUVR between LBD and healthy controls. However, we did observe that entorhinal cortex SUVRs were not significantly different in LBD participants on the basis of Aβ positivity, unlike in healthy controls—a dissociation supporting the idea that early tau deposition within medial temporal lobe may behave differently in the context of synucleinopathy.

Many studies of tau PET in large cohorts of cognitively unimpaired older adults and impaired individuals with Alzheimer’s disease have found that greater tau PET in the temporal lobe is associated with memory performance.^51,52^ In this study of participants with LBD, we observed no significant association with memory for tau in entorhinal cortex or inferior temporal lobe. However, in posterior cortical regions previously reported to show elevated tau signal in LBD,^14-16^ we observed associations such that individuals with greater tau PET uptake performed worse both on tests of executive function and attention/working memory. These relationships between posterior cortical tau PET SUVR and cognition converge with previously reported associations between tau PET in parietal lobe and fluency scores^18^ and associations with global cognition in impaired participants with LBD.^14,19^ Despite associations with cognition that we observed in precuneus and lingual gyrus, it is worth noting that tau PET signal in these regions was not actually elevated in participants with LBD relative to controls. Further research and postmortem validation should determine to what extent the ^18^F-PI-2620 PET signal in these regions is comprised of binding to tau neurofibrillary tangles versus off-target binding to other relevant disease processes, such as iron deposition.^53^ Furthermore, the grouping of individual neuropsychological test scores within discrete cognitive domains (i.e. executive function) is not universally agreed upon, and it will be important for future work to examine how tau PET binding is related to other composite scores of cognitive function in LBD, particularly executive function, working memory and attention.

Binding to neuromelanin in substantia nigra is a feature of several, if not all, tau PET radiotracers that are currently widely used (^18^F-flortaucipir, ^18^F-MK6240, ^18^F-RO948 and ^18^F-PI-2620).^21,22^ We found that ^18^F-PI-2620 SUVR within substantia nigra was reduced in Parkinson’s disease, replicating previous studies of ^18^F-flortaucipir showing differences in substantia nigra signal in Parkinson’s disease^12,25^ and Parkinson’s disease dementia.^18^ This reduction in PET signal within substantia nigra is thought to reflect the loss of dopaminergic neuronal cell bodies, a hallmark of Parkinson’s disease progression.^24^ Accordingly, we found that the degree of substantia nigra signal reduction was associated with severity of motor impairment as measured by the MDS-UPDRS-III exam in an off-medication state across the entire LBD group. However, there was overlap in substantia nigra SUVR across clinical groups, and many participants with Parkinson’s disease displayed substantia nigra SUVR within the range of healthy controls. Additionally, several participants with DLB had normal substantia nigra SUVR despite MDS-UPDRS-III scores indicating motor impairment. Future work should determine to what extent substantia nigra tau PET uptake could serve as a biomarker of dopaminergic neuron loss across clinical phenotypes, which could meet the current need for novel biomarkers of dopaminergic loss and Alzheimer’s disease co-pathology.^54^

This study has several limitations. First, the assessment of amyloid status differed across participants based on the availability of ^18^F-florbetaben PET, CSF and medical records. However, we did have ^18^F-florbetaben PET images available for 38 of the 43 participants with LBD, ensuring that amyloid information within the LBD group was consistent. Second, only cross-sectional ^18^F-PI-2620 PET data were available. Longitudinal studies of ^18^F-flortaucipir in participants with LBD have suggested that cortical tau accumulates in DLB faster than in healthy controls,^55^ but not in Parkinson’s disease.^56^ Future work should determine how ^18^F-PI-2620 PET signal changes in LBD over time. Our study utilized simultaneous PET/MRI, which may introduce bias to PET reconstruction via attenuation correction relative to the gold standard of PET/CT. We also did not utilize partial volume correction. Future work examining different partial volume approaches to tau PET data in the context of LBD are warranted to better understand subtle tau PET elevations in this group. Because of the small sample size and expected small effect sizes, we did not correct our analyses for multiple comparisons. We made efforts to limit the number of comparisons by focusing on four a priori brain ROIs justified by past literature. Finally, while this study sample is one of the largest LBD cohorts to be studied with tau PET imaging, there were small numbers of participants in each diagnostic category, limiting interpretation of ^18^F-PI-2620 in Parkinson’s disease versus DLB, or across cognitive status groups.

In conclusion, we characterized patterns of ^18^F-PI-2620 tau PET binding in LBD and found regionally specific associations with cognition and motor impairment. These results offer the possibility that ^18^F-PI-2620 images carry information about both cortical tau deposition and substantia nigra dopaminergic neuron loss, relevant for assessing Alzheimer’s disease and LBD comorbidity *in vivo*.

## Data Availability

Anonymized data will be made available on request to qualified researchers who have Stanford University’s institutional review board approval and a Data Usage Agreement.
